# Polymorphisms in *MICA*, but not in *DEPDC5*, *HCP5* or *PNPLA3*, are associated with chronic hepatitis C-related hepatocellular carcinoma

**DOI:** 10.1038/s41598-017-10363-5

**Published:** 2017-09-19

**Authors:** Hoang Hai, Akihiro Tamori, Le Thi Thanh Thuy, Kanako Yoshida, Atsushi Hagihara, Etsushi Kawamura, Sawako Uchida-Kobayashi, Hiroyasu Morikawa, Masaru Enomoto, Yoshiki Murakami, Norifumi Kawada

**Affiliations:** Department of Hepatology, Osaka City University Graduate School of Medicine, Osaka, Japan

## Abstract

Recently, the *MICA* rs2596542 and *DEPDC5* rs1012068 variants in Japanese individuals as well as the *HCP5* rs2244546 and *PNPLA3* rs738409 variants in European individuals have been found associated with hepatocellular carcinoma (HCC). The present study determined which single nucleotide polymorphism (SNP) is the most predictive for developing hepatitis C virus (HCV)-related HCC in a Japanese cohort. Of the 4 SNPs analysed, only the *MICA* genotypes were significantly associated with development of HCC (*p* = 0.0185). The major (MA), hetero (HE), and minor (MI) genotypes occurred in 40%, 41%, and 19% of HCC patients and in 43%, 47%, and 10% of non-HCC patients, respectively. Interestingly, the *MICA* genotype was significantly correlated with *MICA* mRNA and soluble protein levels. In patients older than 70 years, the MI genotype was significantly associated with HCC development. In addition, the MI genotype was related to HCC development when the platelet count range was 10–15 × 10^4^/μL, corresponding with the fibrosis stage; but not when the range was less than 10, indicating advanced fibrosis; or greater than 15 × 10^4^/μL, as mild fibrosis. Thus, polymorphisms in *MICA*, but not in *DEPDC5, HCP5* or *PNPLA3*, are associated with HCC development in Japanese patients with chronic HCV infection.

## Introduction

An estimated 130–170 million people worldwide are infected with hepatitis C virus (HCV), and new HCV infections continue to occur^1^. HCV infection is a major cause of liver cirrhosis and hepatocellular carcinoma (HCC)^2^. HCC is the seventh most common type of cancer and the third leading cause of cancer-related deaths worldwide. Approximately 700,000 people die annually from the disease^3^. An estimated 885,000 HCV carriers are Japanese individuals between 16 and 69 years old, and 81% of the 33,000 deaths caused by HCC are infected with HCV^4^.

Although the risk factors for developing HCC, such as hepatitis viruses, aflatoxin B1, heavy alcohol intake and non-alcoholic fatty liver disease^5^, have been well studied, much less is known about host genetic factors. Recently, two independent genome-wide association studies (GWASs) have identified variants associated with HCC in Japanese individuals with chronic HCV (CHC) infection. An intronic single nucleotide polymorphism (SNP) in the *DEPDC5* locus on chromosome 22 is associated with HCC risk^6^, and another SNP, rs2596542, which is located 4.7 kb upstream of the major histocompatibility complex (MHC) class I-related chain A (*MICA*) gene, is also associated with HCC^7^. In Europe, two additional SNPs have been identified as susceptibility loci for HCV-associated HCC, *HCP5* rs2244546 and *PNPLA3* rs738409^8–10^. However, these four SNPs have been studied independently and have not yet been validated within a single cohort. Therefore, we sought to determine which SNPs were predictive of the development of HCV-related HCC in our cohort.

## Results

### Patient profiles and treatment outcomes

The genotype distributions of *MICA, DEPDC5, HCP5*, and *PNPLA3* SNPs in both the HCC and non-HCC groups were in Hardy-Weinberg equilibrium (HWE), as determined with the HWE test. The characteristics of the 717 patients with CHC (349 men and 368 women) are shown in Table 1. All patients were infected with HCV with a viral load >5.0 copies/mL. Significant differences between the HCC (n = 142) and non-HCC groups (n = 575) were observed in age, sex, aspartate transaminase (AST), alanine transaminase (ALT), platelet count, percentage of prothrombin time (PT%), albumin, and AFP (*p* < 0.0001) but not in HCV viral load or the *IL28B* or *ITPA* SNP. The mean age of the patients with HCC was significantly greater than that of the patients without HCC (67 vs. 59 years old). Seventy percent of patients with HCC were male, a value significantly greater than the 43% observed among patients without HCC. AST and ALT levels were significantly higher in the HCC group than in the non-HCC group (69 ± 41 and 67 ± 43 IU/L vs. 49 ± 34 and 55 ± 49 IU/L, respectively). The platelet counts, PT%, and albumin levels in patients with HCC were significantly lower than those in patients without HCC (12.3 ± 6.1 × 10^4^/μL, 88.0 ± 17.5% and 3.8 ± 0.4 g/dL vs. 16.7 ± 6.0 × 10^4^/μL, 99.1 ± 16.2% and 4.2 ± 1.8 g/dL, respectively). Moreover, the mean levels of the tumour markers AFP and PIVKA-II were 630 ng/mL and 2,098 mAU/mL, respectively, in the HCC group, which were significantly higher than those in the non-HCC group (11 ng/mL and 23 mAU/mL, respectively).

**Table 1:** Clinical characteristics of patients in the screening cohort^a^.

Parameter	HCC (n = 142)	Non-HCC (n = 575)	*p*-value
Age (years)	67 ± 9	59 ± 13	<0.0001
Sex (female/male)	42/100	326/249	<0.0001
HCV viral load (log copies/mL)	6.0 ± 1.0	6.2 ± 1.0	0.0718
CH/LC	54/88	487/88	<0.0001
AST (IU/L)	69 ± 41	49 ± 34	<0.0001
ALT (IU/L)	66 ± 43	55 ± 49	<0.0001
Platelets (×10^4^/µL)	12.3 ± 6.1	16.7 ± 6.0	<0.0001
Albumin (g/dL)	3.8 ± 0.4	4.2 ± 1.8	<0.0001
PT%	88 ± 18	99 ± 16	<0.0001
AFP (ng/mL)	630 ± 4357	11 ± 24	<0.0001
PIVKA-II (mAU/mL)	2098 ± 9992	23 ± 43	<0.0001
*IL28B* rs8099917 (TT/TG/GG)^b^	96/34/1	391/143/12	0.5660
*ITPA* rs1127354 (CC/CA/AA)^b^	94/35/2	406/130/9	0.7309

^a^Continuous variables are shown as the mean ± SD.

^b^11 HCC and 30 non-HCC samples were lost to examine the *IL28B* and *ITPA* SNPs, respectively.

### Association between the risk allele of SNPs in 4 genes and the development of HCC in patients with CHC

**Figure 1:**
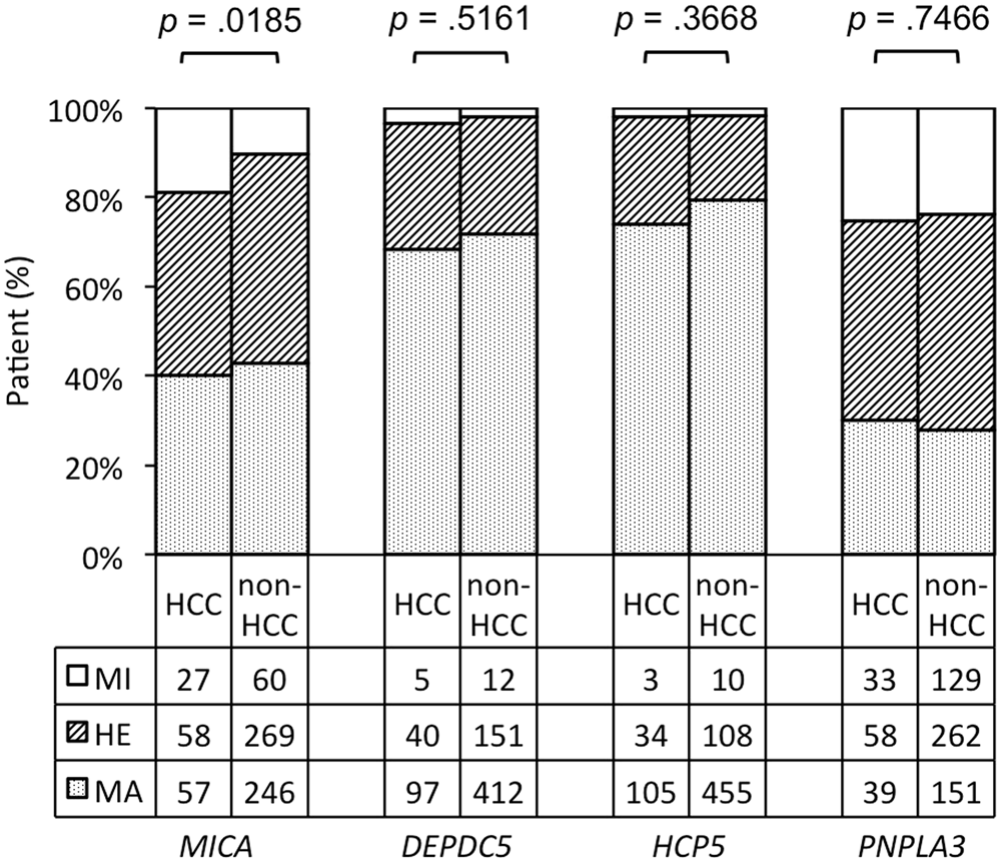
Genotypes of *MICA*, *DEPDC5*, *HCP5*, and *PNPLA3* in patients with or without HCC. The vertical axis shows the percentage of each genotype, and the data table shows the number of samples tested in each group.

**Figure 2:**
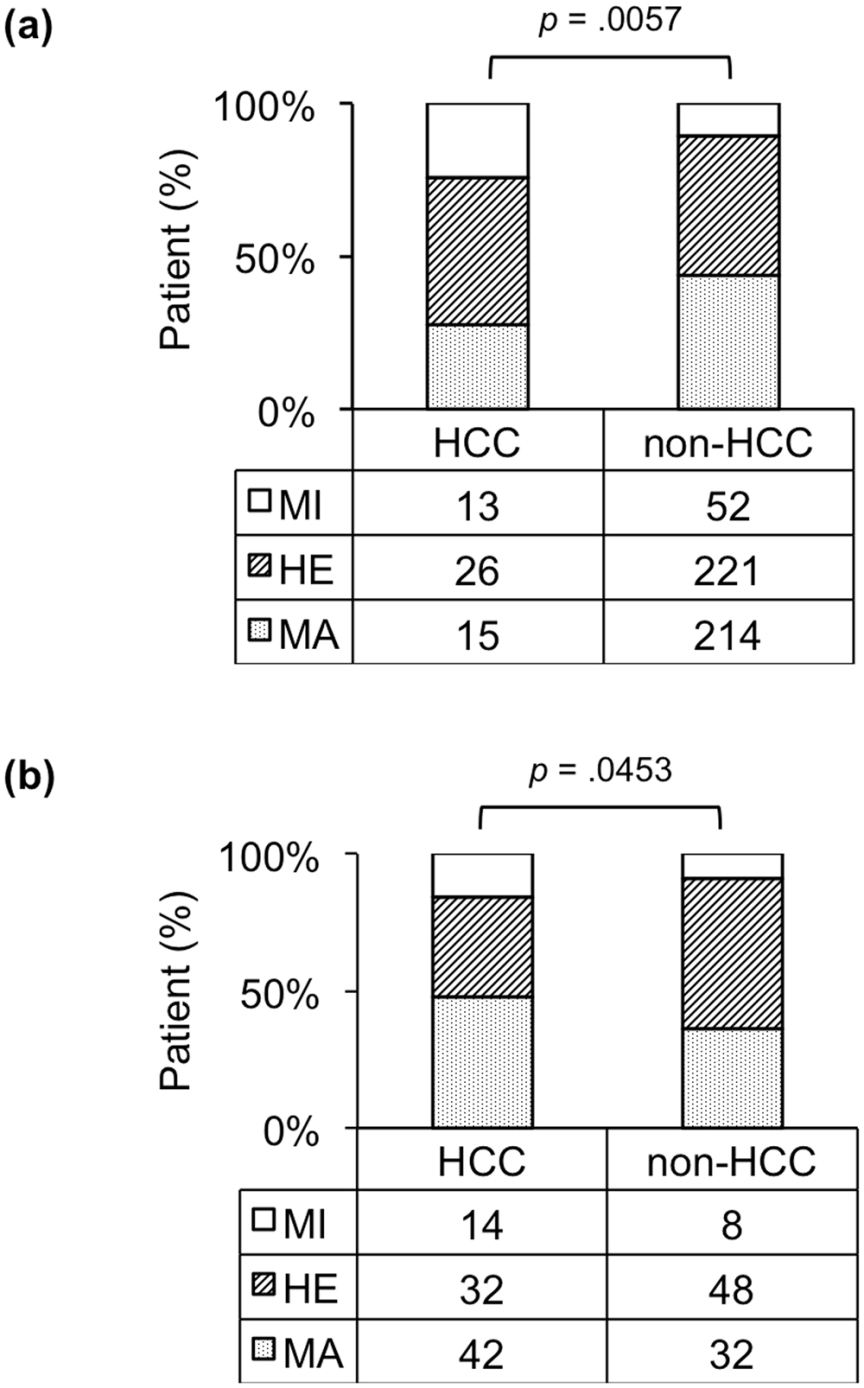
*MICA* genotype and HCC development. A group of patients with background of CHC (**a**, n = 541) or cirrhosis (**b**, n = 176) is shown.

To investigate the association between the *MICA* SNP and HCC development, we genotyped 717 samples by using TaqMan SNP genotyping assays. Major (MA), hetero (HE), and minor (MI) genotypes were present in 57 (40%), 58 (41%), and 27 (19%) patients with HCC, respectively, and in 246 (43%), 269 (47%), and 60 (10%) patients without HCC, respectively, indicating a significant association between the *MICA* genotype and HCC development in patients with CHC (*p* = 0.0185, Fig. 1). The MI allele frequencies (MAFs) in the HCC and non-HCC groups were 0.394 and 0.338, respectively. In addition to the association of all 717 patients, a significant association also was observed in sub-cohorts of 541 chronic (*p* = 0.0057, Fig. 2a) and 176 cirrhosis patients (*p* = 0.0453, Fig. 2b).

Furthermore, a validation study was performed in 638 CHC patients including 115 those with HCC and 523 without HCC (Supplementary Table S1). Interestingly, the *MICA* SNP again was found to significantly associate with HCC development in patients with CHC (*p* = 0.0131). The MA, HE, and MI genotypes were present in 48 (41.8%), 45 (39.1%), and 22 (19.1%) patients with HCC, respectively, and in 250 (47.8%), 223 (42.6%), and 50 (9.6%) patients without HCC, respectively (Supplementary Fig. S1).

In addition to the *MICA* SNP, we also analysed three other reported HCV-related HCC SNPs. For the *DEPDC5* SNP, we found MA, HE, and MI genotypes in 97, 40, and 5 patients with HCC, respectively, and in 412, 151, and 12 patients without HCC, respectively (*p* = 0.5161); the MAFs were 0.18 and 0.15 for the HCC and non-HCC groups, respectively. For the *HCP5* SNP, we observed the MA, HE, and MI genotypes in 105, 34, and 3 patients with HCC, respectively, and in 455, 108, and 10 patients without HCC, respectively (*p* = 0.3668). Finally, the MA, HE, and MI *PNPLA3* genotypes were observed in 39, 58, and 33 patients with HCC, respectively, and in 151, 262, and 129 patients without HCC, respectively (*p* = 0.7466, Fig. 1).

### Independent factors related to HCC development

*MICA* SNP and variables with *p* values < 0.0001 in the univariate analysis (Table 1) were subjected to logistic regression analysis. These variables including age, sex, albumin, prothrombin time, AFP, AST, ALT, PIVKA-II concentration, platelets, and genotype of the *MICA* SNP were categorized and used to analyse associations with binary outcomes (HCC or non-HCC). Logistic regression analysis indicated that age (older than 65 years old), male sex, albumin ≤ 4 g/dL, prothrombin time ≤70%, AFP concentration ≥ 20 ng/mL, PIVKA-II concentration ≥ 40 mAU/mL, and minor genotype of the *MICA* SNP were independent factors that were significantly associated with HCC development (Table 2).

**Table 2:** Logistic regression analysis of independent factors related to HCC development

Variable	OR (95% CI)	*p*-value
Age (≥66 years vs.<65 years)	3.68 (2.02–6.71)	<0.0001
Sex (male vs. female)	4.01 (2.21–7.27)	<0.0001
AST (≥40 IU/L vs. <40 IU/L)	1.04 (0.47–2.32)	0.9243
ALT (≥40 IU/L vs. <40 IU/L)	1.52 (0.71–3.23)	0.2797
Plt (≤15 × 10^4^/µL vs. >15 × 10^4^/µL)	1.26 (0.67–2.36)	0.4719
Alb (≤4 g/dL vs. >4 g/dL)	2.84 (1.49–5.42)	0.0015
PT% (≤70 vs. >70)	3.90 (1.32–11.56)	0.0140
AFP (≥20 ng/mL vs. <20 ng/mL)	4.31 (2.19–8.49)	<0.0001
PIVKA-II (≥40 mAU/mL vs. <40 mAU/mL)	11.44 (5.32–24.57)	<0.0001
*MICA* genotypes (minor vs. non-minor)	4.47 (2.04–9.80)	0.0002

### The MICA SNP was correlated with MICA mRNA and soluble protein levels

**Figure 3:**
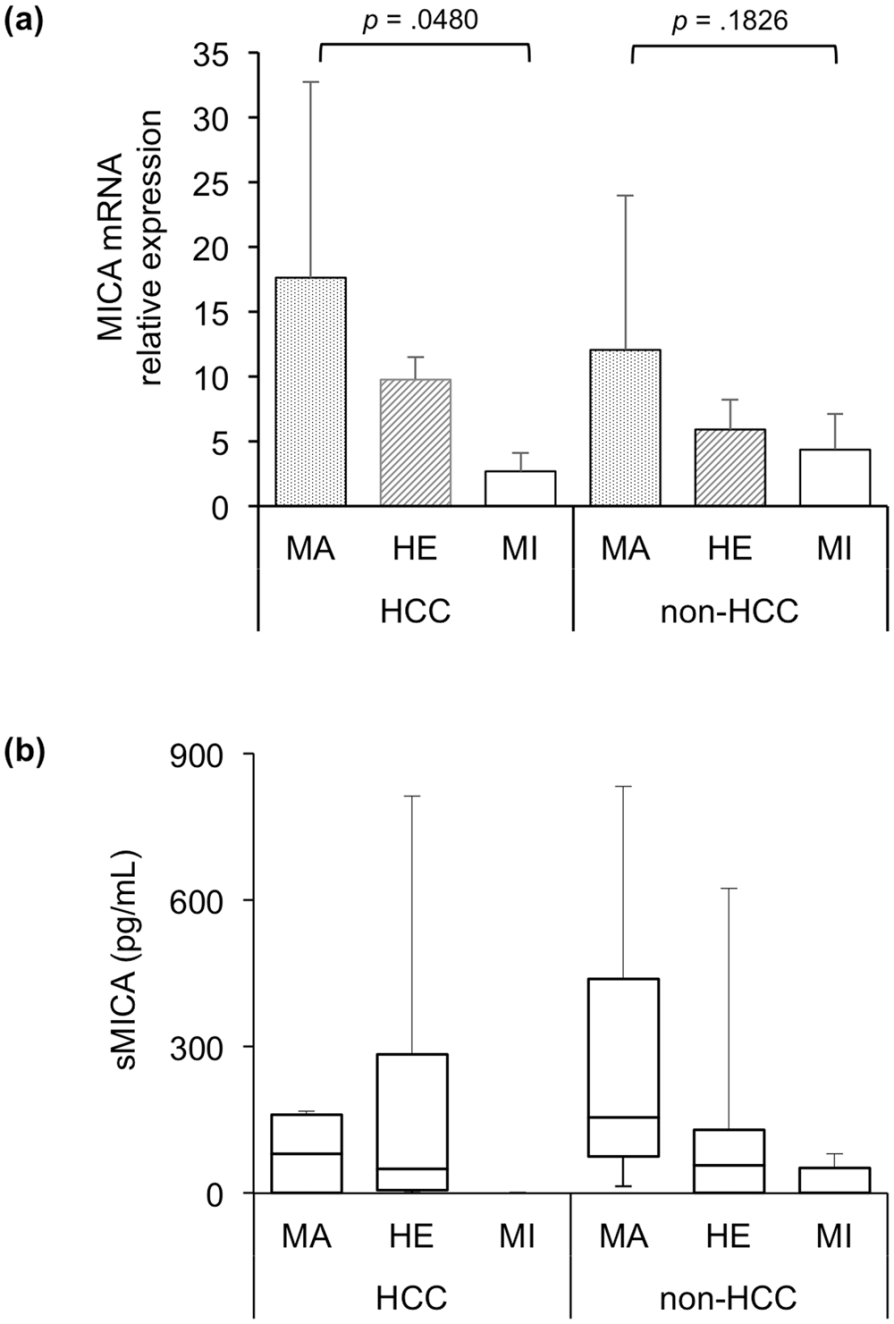
*MICA* SNP genotypes with mRNA and soluble protein levels. *MICA* relative expression levels were determined by real-time quantitative RT-PCR using paired-tumour (HCC) and adjacent non-tumour liver tissues (non-HCC) from 21 HCV patients (**a**). Note that mRNA expression of *MICA* MI genotype was significantly down regulated in HCC group. N = 11, 5, and 5 in MA, HE, and MI genotypes, respectively. Median protein levels in HCC or non-HCC samples with MA, HE, and MI genotypes are shown as horizontal lines inside the box of interquartile range (**b**). The whiskers are the maximum and minimum values. We assessed the difference in the median values among genotypes by using Kruskal-Wallis tests (*p* = 0.1284 in HCC group; *p* = 0.0498 in non-HCC group).

Because the MI genotype of the *MICA* SNP was associated with a high risk of HCC development, we assessed whether rs2596542 was correlated with MICA expression in patients with HCV-related HCC. We examined the transcription level of *MICA* using paired HCC and adjacent non-tumour liver tissues from 21 individuals with HCV. As shown in Fig. 3a, real-time quantitative PCR assays revealed a significant decreased mRNA expression of *MICA* MI genotype in tumour tissues compared to MA genotype (*p* = 0.048). Furthermore, sMICA levels were measured in 36 serum samples (n = 6 of each genotype from patients with or without HCC) by using ELISAs. The results showed that in the HCC samples, median protein levels of sMICA from patients with the MA, HE, and MI genotypes were 80, 50, and 0 pg/mL, respectively (Fig. 3b). Although these protein levels tended to be higher in MA and HE, and lower in MI genotypes, they were not significantly different (*p* = 0.1284 by Kruskal-Wallis test). However, interestingly, in the non-HCC group, the sMICA levels were significantly correlated with its genotypes (*p* = 0.0498 by Kruskal-Wallis test, Fig. 3). These results suggest that MICA mRNA and protein expression likely correlate with the *MICA* genotype.

### The MICA MI genotype is related to HCC development in patients older than 70 years

**Figure 4:**
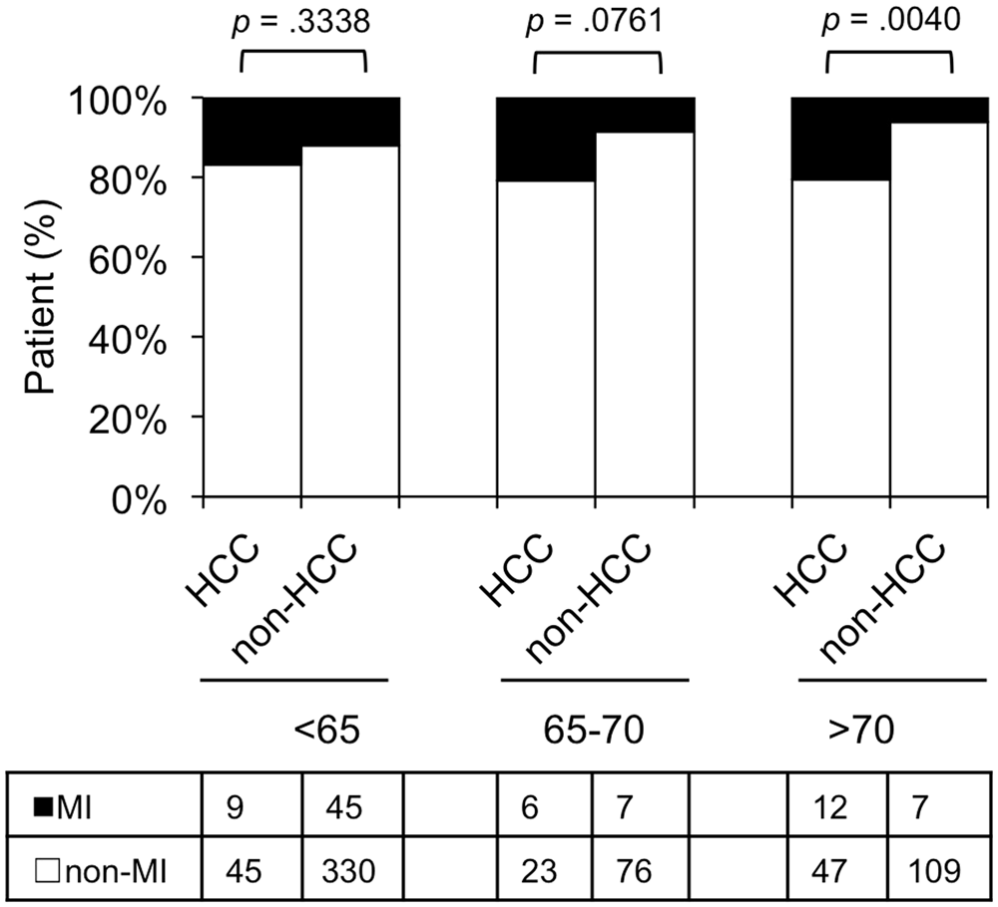
*MICA* rs2596542 MI genotype and the age of patients. Patients with or without HCC were divided into 3 age groups: <65, 65–70, and >70. The vertical axis shows the percentage of each genotype, and the data table shows the number of independent samples tested in each group.

We determined whether the risk allele rs2596542 was related to HCC development when patients were stratified by age. By comparing patients with and without HCC across 3 groups of age (younger than 65 years old, 65–70 years old, and above 70 years old), we found that the MI genotype was significantly associated with HCC development in the subset of patients with CHC above 70 years old (*p* = 0.004, Fig. 4).

**Figure 5:**
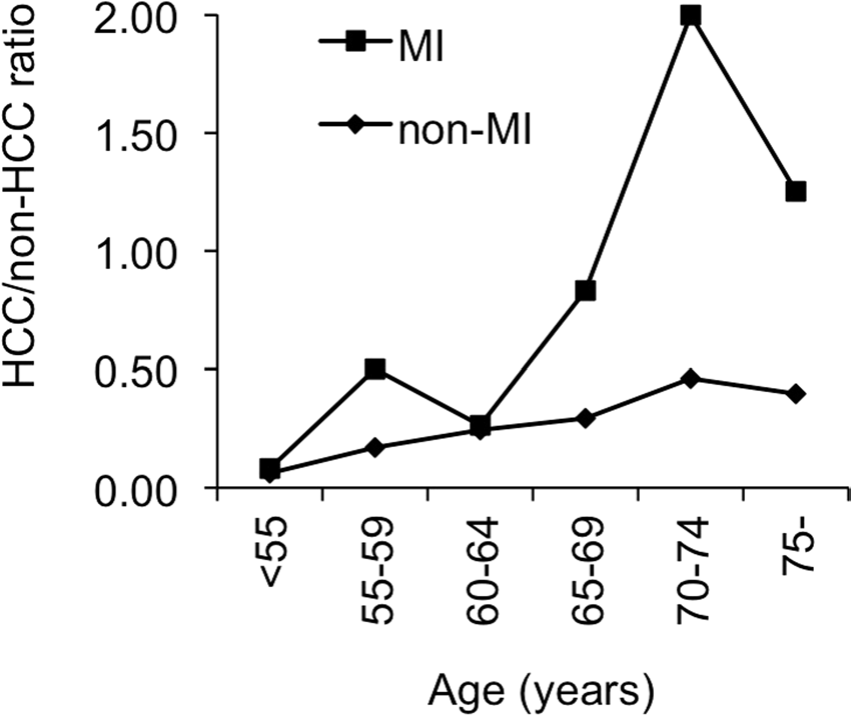
HCC/non-HCC ratio of each 5-year age group with respect to the *MICA* rs2596542. The vertical axis shows the HCC/non-HCC ratio in the MI genotype (■) or non-MI genotype (♦), and the horizontal axis shows age grouped by a 5-year period.

Moreover, we analysed the ratio of patients with HCC to those without HCC with respect to the *MICA* genotypes in 5-year age ranges: younger than 55 years old, 55–59 years old, 60–64 years old, 65–69 years old, 70–74 years old, and above 74 years old. This ratio varied among MI allele carriers from 0.06 to 2.0, which was greater than the range of 0.06 to 0.46 observed among patients with the non-MI allele (Fig. 5).

### The MICA MI genotype is associated with the development of HCC in patients with platelet counts in the range of 10–15 × 10^4^/μL

**Figure 6:**
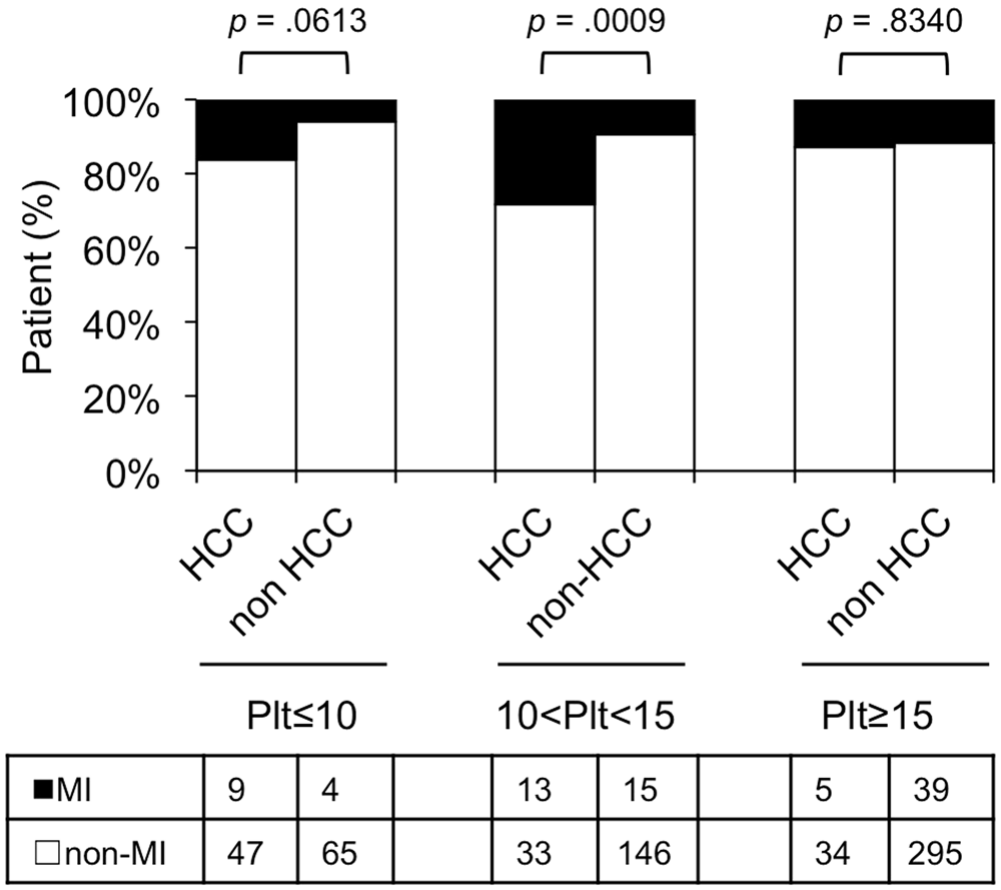
*MICA* SNP MI genotype and platelet counts. The vertical axis shows the percentage of each genotype in HCC or non-HCC patients, and the data table shows the number of independent samples tested in each group of platelet counts. Plt ≤ 10, platelet counts equal or less than 10 × 10^4^/μL blood; 10 < Plt < 15, platelet counts in the range of 10–15 × 10^4^/μL blood; Plt ≥ 15, platelet counts equal or more than 15 × 10^4^/μL blood.

We took advantage of knowing that platelet count reflects stage of liver fibrosis^11^ in which a decrease in platelet count in accordance with severity of fibrosis in CHC patients^12,13^. In this study, we determined the platelet count range at which MI rs2596542 was related to the development of HCC. We found that the *MICA* MI genotype was associated with HCC development when the platelet count was 10–15 × 10^4^/μL, corresponding with the fibrosis stage; but not when the range was less than 10, indicating advanced fibrosis; or greater than 15 × 10^4^/μL, as mild fibrosis (Fig. 6).

## Discussion

Both host genetic and environmental factors affect the progression of liver disease over multiple stages. With respect to host genetic factors, variations in the *IL28B* gene affect the progression of CHC due to HCV exposure^14,15^, and variations in the *ITPA* gene are associated with the outcomes of interferon and ribavirin combination therapy^16^. The genetic factors that play a role in late-stage liver disease remain controversial, although several studies have shown that variants in *MICA, DEPDC5, HCP5* and *PNPLA3* affect HCC development^6–8,17^. The SNPs listed above were associated with HCV-related HCC in four separate cohorts. This study is the first to evaluate the association between these SNPs and HCV-related HCC in a unique cohort from Japan. We found a strong association between *MICA* SNP and HCC development, particularly in older patients or patients with fibrosis.

In our cohort, the MI allele A of rs2596542 in *MICA* was determined to be a likely risk factor for the development of HCC. This result is consistent with findings from a previous study in Japan, which has reported similar MAFs of 0.398 and 0.333 in HCC and non-HCC groups, respectively^7^. In contrast, Lange *et al*., in the Swiss Hepatitis C Cohort Study (SCCS), have found that the MI allele exerts a protective effect against HCC development^8^. The MI allele A of rs2596542 might be a risk allele in Japanese patients but a protective allele in European patients with HCC. In the SCCS, a lower MAF of 0.241 in patients with HCC and a higher MAF of 0.361 in patients without HCC supported this hypothesis. In addition, the proportion of patients with HCC in our cohort was 20% (142/717), a value much greater than the 3% (64/1924) observed in the SCCS. The association of *MICA* genotype with HCC development was also confirmed in both CHC and LC background patients of our cohort (Fig. 2). Importantly, our findings were validated using another cohort of 638 patients with CHC, which showed the same results with the first cohort (Supplementary Table S1 and Supplementary Fig. S1).

We did not find any associations between the 3 other SNPs tested (i.e., *DEPDC5* rs1012068, *HCP5* rs2244546 and *PNPLA3* rs738409) and the development of HCC in patients with CHC. Although the *DEPDC5* variant was associated with progression to HCC in a previous Japanese cohort^6^, our data did not show this association. The disparity between these results may be due to differences in study design. Miki studied patients over 55 years old, whereas our study enrolled patients regardless of age. This difference resulted in a higher proportion of patients with HCC in Miki’s study (30%) compared with the 20% in our cohort. One additional explanation is that the MAF for the control group from Miki’s study was lower than that in our study (0.10 vs. 0.15, respectively). However, our results are consistent with the most recent report from Europe, which has shown that the *DEPDC5* variant is not associated with HCC^18^. Similarly to the *DEPDC5* SNP, there was no association between the *HCP5* rs2244546 variant and HCC development. Two causes might explain this finding. First, the *HCP5* variant affects HCV-related development of HCC in European but not Japanese patients. Second, as described above, fewer patients with HCC and no MI genotype GG were present in the HCC group of the previous study^8^. Furthermore, the well-known alcoholic and nonalcoholic fatty liver disease-associated variant in *PNPLA3* rs738409^9,19,20^ has recently been shown to affect the development of HCC in European individuals with CHC^9,10,17^. In contrast, Japanese ethnicity may have resulted in no significant association of rs738409 with HCC development in our cohort. In Japan, a study showed that the *PNPLA3* minor genotype is associated with the age at onset of HCC^21^, and another study with a small number of total patients as well as HCC patients has indicated that the *PNPLA3* SNP is indirectly associated with HCC development via serum AFP level^22^. The greater proportion of HCC patients in our cohort might explain the apparent discrepancy. Moreover, Trepo^23^ has noted that the results obtained from European patients with HCV-related HCC remain controversial because 2 other studies have not found a positive relationship between the *PNPLA3* risk allele and HCC development in HCV-infected patients^24,25^. Furthermore, a secondary analysis of the data from the American HALT-C trial has not identified a significant association between rs738409 and HCC development in patients with HCV^26^.

Additionally, MICA mRNA and protein levels were significantly correlated with the *MICA* variants, thus further supporting the association between the *MICA* MI genotype and the risk of HCC development. In normal tissues, MICA expression is relatively low but is elevated in tumour tissues^27^. Here we also found the *MICA* mRNA level were higher in liver tumour tissues compared to non-tumour area, down-regulated in *MICA* HE genotype, and lowest in MI genotype (Fig. 3a). As a natural killer (NK) group 2D ligand, membrane-bound MICA triggers the immune system, thereby resulting in the elimination of target tumour cells via NK and CD8+T cells^28,29^. In our study, which was consistent with the data from another Japanese cohort^7^, patients with HCC carrying the MI allele A of rs2596542 expressed low levels of sMICA protein, which is probably excreted through shedding of membrane-bound MICA protein, thus eliminating NK and CD8 + cell activity in viral-infected cells. Consequently, risk allele carriers are more likely to develop HCC from CHC. This same sMICA trend was found in patients with CHC, whose sMICA levels were 0 pg/mL in individuals with the MI genotype but gradually increased in those with the HE and MA genotypes. These findings were similar to those of Kumar, who has reported median sMICA levels of 0, 69, and 65 pg/mL for the MI, HE and MA genotypes, respectively, and levels of 0, 65 and 78 pg/mL for patients without HCV, with CHC, and with HCC, respectively^7^. These results suggest that sMICA levels are elevated during the chronic stage of disease, and this condition has the potential to develop into HCC.

Hepatic fibrosis is a major risk factor for HCC in patients with CHC; however, age is another important factor because HCC can develop in older patients with CHC and mild fibrosis. To determine the age threshold at which the MI genotype of *MICA* is most related to HCC development, we compared the MI genotype in patients with and without HCC with respect to age. Our results suggest that older patients (>70 years old) with HCC exhibit an increased carriage of *MICA* MI. Other studies have used age cutoffs of 60 or 65 years old to analyse the association between age and HCC development^6,7^. Moreover, our data suggest that *MICA* is a protective factor against HCC development in older patients because the *MICA* non-MI genotype predominated in older patients without HCC compared with those with HCC. Notably, the ratio of patients with HCC to those without HCC with the rs2596542 MI allele was higher than that in patients without the MI allele across all 5-year age ranges. In addition, with increasing age, the slope of the MI allele carriers was steeper than that of the non-MI allele carriers. Developing HCC was significantly associated with *MICA* and *DEPDC5* SNPs in two separate studies; Kumar *et al* ^7^. did not mention the ratio of HCC to non-HCC in their study of the *MICA* SNP, whereas this trend corroborates findings of Miki’s study of the *DEPDC5* SNP^6^. These results confirmed that HCC develops more often in older patients and that the risk allele is also associated with the development of HCC in older patients.

There is a reverse correlation between peripheral platelet count and liver fibrosis stage, in which the more platelet count the less liver fibrosis stage^11^. In our cohort, the prevalence of *MICA* genotypes in patients with HCC differed from that in patients without HCC with a platelet count of ≤10 × 10^4^/µL. Furthermore, the difference became significant in patients with a platelet count in the range of 10–15 × 10^4^/µL. No differences were found in patients with platelet counts greater than 15 × 10^4^/µL. Since the platelet count in the range of 10–15 × 10^4^/µL corresponds with the fibrosis stage^13,30^, our data suggest that the MI *MICA* genotype may associate with HCC in patients with fibrosis.

As with previous studies of these 4 SNPs, the present study has limitations. First, these studies were retrospective, cross-sectional analyses. With a longer follow-up period, more patients with continuous HCV infection would be likely to develop HCC, and patients’ genetic backgrounds are not the only predictor of HCC development. Also, there was no information about past treatment for CHC in the present study. In addition, sMICA levels were examined in only 36 patients. However, the results were consistent with *MICA* mRNA level in HCC and non-tumour tissues.

## Conclusion

The *MICA* rs2596542 genotype was correlated with *MICA* mRNA and protein levels, and the MI genotype of *MICA* was associated with an increased risk of HCC development in patients with HCV infection. This finding was particularly true for patients older than 70 years, even those with fibrosis. Our data suggest that *MICA* plays an important role in the development of HCC in patients with CHC.

## Patients and Methods

### Patients

This study was a cross-sectional analysis. A total of 717 patients were recruited at Osaka City University Hospital between December 2004 and December 2013. All patients had either a viral load of >10^5^ IU/mL according to the COBAS AMPLICOR HCV Monitor test, version 2.0 (Roche Diagnostics, Branchburg, NJ, USA), or a viral load of >5 log copies/mL as determined by the COBAS TaqMan HCV test (Roche Diagnostics). HCC was diagnosed at the conclusion of the data collection in December 2013. All patients provided written informed consent, and all methods were carried out in accordance with the ethical guidelines of the 1975 Declaration of Helsinki. All experimental protocols were approved by the ethical committee of Osaka City University, Graduate School of Medicine (approval No. 1646).

The exclusion criteria included a history or evidence of a serious chronic or poorly controlled medical or psychiatric condition and infection with human immunodeficiency virus or hepatitis B virus. Patients with autoimmune liver diseases, primary biliary cirrhosis, and heavy alcoholic habits were also excluded.

We next recruited CHC patients from May 2013 to April 2017 for a replication study. A total of 638 patients were further analysed as a validation cohort.

### Surveillance and diagnosis of HCC and analyse related factors

For HCC surveillance, patients underwent ultrasonography, computerised tomography (CT) and/or magnetic resonance imaging (MRI). If screened patients was suspected HCC, tumour biopsy, dynamic CT, dynamic MRI, contrast enhanced ultrasonography and hepatic angiography were examined. The following factors were analysed to determine whether they were related to the development of HCC: patient age, sex, and pre-treatment haematological and biochemical parameters, such as platelet counts, ALT levels, AST levels, HCV viral load, alpha-fetoprotein (AFP), albumin, and protein induced by vitamin K absence-II (PIVKA-II). Approximately 1–2% of the patients had missing data for the haematology and biochemical parameters.

### SNP genotyping

We examined the genetic polymorphisms *IL28B* rs8099917 (T/G)*, ITPA* rs1127354 (C/A), *DEPDC5* rs1012068 (T/G), *HCP5* rs2244546 (C/G), *MICA* rs2596542 (G/A), and *PNPLA3* rs738409 (C/G) in patients who consented to a genome analysis. Whole blood was collected from all patients and was centrifuged to separate the buffy coat. Genomic DNA was extracted from the buffy coat using a QIAamp^®^ DNA Blood Midi Kit (QIAGEN GmbH, QIAGEN Strasse 1, 40724 Hilden, Germany). Genetic polymorphisms of SNPs were genotyped using (1) TaqMan SNP Genotyping Assays via a 7500 Fast Real-Time PCR System (Applied Biosystems, Foster City, CA, USA) and (2) direct sequencing. Approximately 10% of the samples were also randomly genotyped via direct sequencing to confirm the genotypes. A fragment of *MICA* was amplified via polymerase chain reaction (PCR) using the following primers: forward, 5′-CCTCAGGTTATCTGCCTGCCA-3′; reverse, 5′-CATCTTATTGGGACATACTTTGCAT-3′. The primers for *DEPDC5* were Fw-5′- AGTCGGTTTTCAGTGTGGTGG-3′ and Rv-5’-CAGGTTCAACTCTCAGAGCCATC-3′; those for *HCP5* were Fw-5′-TCACCTTCTGCCGTGATTCT-3′ and Rv-5′-GGAGCTTTGCAGGAACTAGC-3′; and those for *PNPLA3* were Fw-5′-TGTGAGCACACTTCAGAGGC-3′ and Rv-5′-TGGGTCAAAAGAACGGGGAA-3′. PCR was performed in a total volume of 20 μL with 1 × Premix Ex Tag (TaKaRa Bio Inc., Otsu, Shiga, Japan), 300 nM of each primer and 100 ng of genomic DNA. The PCR protocol was performed at 94 °C for 10 min followed by 35 cycles of 94 °C for 30 s, 62 °C for 30 s and 72 °C for 1 min, with a final extension at 72 °C for 7 min. PCR products were sequenced bi-directionally using a BigDye Terminator v3.1 Cycle Sequencing Kit and an 3130XL Genetic Analyser (Applied Biosystems, Foster City, CA, USA). Genotyping of *IL28B* and *ITPA* SNPs were performed as previously described^16^. Direct sequencing results were completely matched with TaqMan SNP genotyping assay. The ethical committee of our university permitted the genotyping analysis (approval No. 1871).

### Quantitative Real-time PCR

A total of 21 paired primary HCC and adjacent non-tumour tissues were examined. Total RNA were extracted from these liver tissues using Direct-zol™ RNA Kits, (Zymo research, CA, USA). cDNAs were synthesised using 1 μg total RNA, a ReverTra Ace qPCR RT Kit (Toyobo, Osaka, Japan) and oligo(dT)_12–18_ primers according to the manufacturer’s instructions. Gene-specific oligonucleotide primers for *MICA* as the following: *MICA*-forward: 5′-CCTTGGCCATGAACGTCAGG-3′; *MICA*-reverse: 5′-CCTCTGAGGCCTCGCTGCG-3′; Gene-specific oligonucleotide primers for *GAPDH* as: *GAPDH*-forward: 5′-GCACCGTCAAGGCTGAGAAC-3′; *GAPDH*-reverse: 5′-TGGTGAAGACGCCAGTGGA-3′. Gene expression was measured by real-time quantitative RT-PCR using the cDNAs, SYBR qPCR Mix Reagents (Toyobo) and above primers with an ABI Prism 7500 Fast Real-Time PCR System (Applied Biosystems, Foster, CA). The *GAPDH* level was used to normalize the relative abundance of mRNAs.

### Measuring soluble MICA (sMICA) protein levels

We randomly chose a group of 6 patients with each *MICA* genotype from either the HCC or non-HCC group for further analysis of soluble MICA levels. The characteristics of these 36 patients were present in Supplementary Table S2. Serum was collected after the blood was allowed to clot. The clot was removed by centrifugation, and sMICA level in the resulting supernatants from 36 samples were quantified using a RayBio Human MICA ELISA Kit as described in the manufacturer’s instructions (RayBiotech, Norcross, GA, USA). We assessed whether the soluble MICA levels decreased from the major to minor genotype in either group of HCC or non-HCC. We used the Kruskal-Wallis test for analysis.

### Statistical analyses

All data analyses were conducted using the JMP program, version 9.0 (SAS Institute, Cary, NC, USA). Individual between-group characteristics were evaluated using Wilcoxon’s two-sample test for continuous variables or Fisher’s exact test for categorical variables. Hardy-Weinberg equilibrium (HWE) was tested to assess the quality of the SNP data. To control for confounders, variables exhibiting *p* values < 0.0001 in univariate analysis were subjected to logistic regression analysis^31^. A *p* value of < 0.05 was considered to be statistically significant.

## Electronic supplementary material

Supplementary Information
