# Supplementary material for: Polymorphisms in MICA, but not in DEPDC5, HCP5 or PNPLA3, are associated with chronic hepatitis C-related hepatocellular carcinoma
Source: Sci Rep. 2017 Sep 19;7:11912. doi: 10.1038/s41598-017-10363-5 (PMC5605683; doi:10.1038/s41598-017-10363-5)
Supplement: Supplementary file 1 — Supplementary Information [file 41598_2017_10363_MOESM1_ESM.pdf]

Polymorphisms in *MICA*, but not in *DEPDC5*, *HCP5* or *PNPLA3*, are associated with chronic hepatitis C-related hepatocellular carcinoma

Hoang Hai, Akihiro Tamori*, Le Thi Thanh Thuy, Kanako Yoshida, Atsushi Hagihara, Etsushi Kawamura, Sawako Uchida-Kobayashi, Hiroyasu Morikawa, Masaru Enomoto, Yoshiki Murakami, Norifumi Kawada

Department of Hepatology, Osaka City University Graduate School of Medicine, Osaka, Japan

***Corresponding author**
Akihiro Tamori, MD & PhD
Department of Hepatology, Osaka City University Graduate School of Medicine
1-4-3, Asahimachi, Abenoku, Osaka 545-8585, Japan.
Tel: +81-6-6645-3905
Fax: +81-6-6646-1433
E-mail: atamori@med.osaka-cu.ac.jp

Supplementary Table S1. Clinical characteristics of patients in the validation cohort

Parameter	HCC (n = 115)	Non-HCC (n = 523)	*p*-value
Age (years)	70 ± 8	60 ± 13	<0.0001
Sex (female/male)	42/73	288/235	0.0004
HCV viral load (log copies/mL)	6.0 ± 0.8	6.0 ± 1.0	0.5151
CH/LC	47/62	479/44	<0.0001
AST (IU/L)	73 ± 49	56 ± 56	<0.0001
ALT (IU/L)	58 ± 36	58 ± 59	0.0192
Platelets ($\times 10^4/\mu L$)	11.5 ± 5.0	17.9 ± 7.1	<0.0001
Albumin (g/dL)	4.2 ± 6.9	4.2 ± 3.1	<0.0001
PT%	84 ± 15	95 ± 18	<0.0001
AFP (ng/mL)	2770 ± 14985	25 ± 284	<0.0001
PIVKA-II (mAU/mL)	3062 ± 21595	595 ± 7445	<0.0001
IL28B rs8099917 (TT/TG/GG)	82/31/1	360/143/8	0.5770
ITPA rs1127354 (CC/CA/AA)	59/31/3	315/105/4	0.1139

Supplementary Table S2. Characteristics of patients available for sMICA level

Parameter	All patients (n = 36)	HCC (n = 18)	Non-HCC (n = 18)
Age (years)	61 ± 11	63 ± 9	59 ± 13
Gender (female/male)	15/21	2/16	13/5
CH/LC	18/18	9/9	9/9
HCV RNA (log copies/mL)	5.9 ± 1.4	6.2 ± 1.4	5.7 ± 1.4
AST (IU/L)	64.1 ± 63.3	74.4 ± 70.7	53.9 ± 53.0
ALT (IU/L)	58.2 ± 48.5	64.7 ± 34.3	51.6 ± 58.7
Plt ($\times 10^4/\mu L$)	13.1 ± 5.4	12.0 ± 4.0	14.3 ± 6.3
Alb (g/dL)	4.1 ± 0.5	4.0 ± 0.5	4.2 ± 0.5
PT%	95.8 ± 21.8	93.3 ± 20.7	99.0 ± 22.9
AFP (ng/mL)	50.1 ± 101.4	80.9 ± 128.4	15.4 ± 32.5
PIVKA- Ⅱ (mAU/mL)	1060.2 ± 4622.0	1864.8 ± 6041.4	25.8 ± 21.2
MICA genotype (MA/HE/MI)	12/12/12	6/6/6	6/6/6

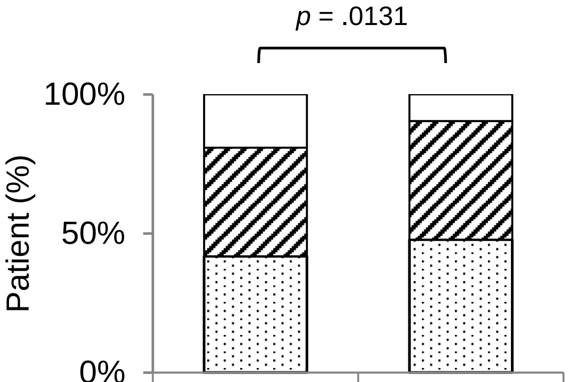

	HCC	non-HCC
MI	22	50
HE	45	223
MA	48	250

Supplementary Figure S1. Genotypes of *MICA* in CHC patients with or without HCC in the validation cohort. The vertical axis shows the percentage of each genotype, and the data table shows the number of samples tested in each group.
